# Paths to Cheminformatics: Q&A with Norberto Sánchez-Cruz and Emma Schymanski

**DOI:** 10.1186/s13321-022-00628-1

**Published:** 2022-08-02

**Authors:** Norberto Sánchez-Cruz, Emma L. Schymanski

**Affiliations:** 1 Instituto de Química, Unidad Mérida, Universidad Nacional Autónoma de México, Carretera Mérida-Tetiz Km. 4.5, 97357 Ucú, Yucatán Mexico; 2 Chemotargets SL, Baldiri Reixac 4, Parc Cientific de Barcelona, 08028 Barcelona, Catalonia Spain; 3 Luxembourg Centre for Systems Biomedicine (LCSB), University of Luxembourg, 6 Avenue du Swing, 4367 Belvaux, Luxembourg

## Introduction by the Editors-in-Chief

The Editors of *Journal of Cheminformatics* outlined their plans for future developments and specific goals for the journal in their recent Editorial [1]. One focus for the upcoming years is the amplification of activities to help create a more inclusive and diverse research and publishing environment. In order to reach this goal, a necessary first step is starting conversations with different individuals from the cheminformatics community, highlighting the various career paths that they have taken, including difficulties they faced and advice they might give to their peers.

With this contribution, we are starting an interview series, each featuring two cheminformatics and/or computational scientists from different career stages and with different backgrounds. For this first round, we invited Norberto Sánchez-Cruz (hereafter referred to as NSC) and Emma L. Schymanski (ELS) who kindly provided insights into their careers and private lives to shed light on challenges, as well as ways to increase diversity and inclusion within our community and research environments.

NSC is Assistant Professor at the Institute of Chemistry, National Autonomous University of Mexico (UNAM). His research is focused on the development, validation, and application of in silico tools to support drug discovery, particularly those related to cheminformatics, molecular modelling, and artificial intelligence. In addition, he maintains a close collaboration with Chemotargets SL, taking part in drug discovery projects within the pharmaceutical industry.

ELS is Associate Professor and head of the Environmental Cheminformatics (ECI) group at the Luxembourg Centre for Systems Biomedicine (LCSB), University of Luxembourg. Her research combines cheminformatics and computational (high resolution) mass spectrometry approaches to elucidate the unknowns in complex samples and relate these to environmental causes of disease. She is involved in and organizes several European and worldwide activities to improve the exchange of data, information and ideas between scientists, including NORMAN-SLE, MassBank, MetFrag and PubChemLite for Exposomics.

## What has been your path to where you are today?

*NSC:* I have spent almost my entire life living in the metropolitan area of Mexico City, where I studied pharmaceutical and biological chemistry (aka medicinal chemistry) in the National Autonomous University of Mexico (UNAM). In the last year of my bachelor's degree, during a research stay at the Institute of Physical Sciences at UNAM, I had my first contact with programming and molecular modelling, which inspired me to start a master’s degree in chemistry at UNAM under the supervision of Dr. Ramón Garduño Juárez and work on the development of statistical potentials to study the energetics of protein folding. After that, I became interested in cheminformatics and drug design thanks to Dr. José Luis Medina Franco, and started my PhD in chemistry under his supervision, also at UNAM, working in the development and validation of computational tools for drug design [2–5]. As part of my PhD, I had the opportunity to carry out two research visits to Barcelona, Spain: the first with the Computational Biology and Drug Design Group at the University of Barcelona (UB) under supervision of Dr. Xavier Barril, followed by another at the Systems Pharmacology Group of the Research Programme on Biomedical Informatics (GRIB) at Hospital del Mar Medical Research Institute (IMIM) under supervision of Dr. Jordi Mestres. Once I completed my PhD, these experiences opened up the opportunity for me to work as a Research Scientist for Chemotargets SL, where I had a closer look to the computational drug discovery pipeline from an industry point of view. Currently, I am starting a position as Assistant Professor in the Institute of Chemistry at UNAM, for which I am very grateful. *ELS:* I grew up and studied chemistry and environmental engineering in Australia (mostly in Perth, Western Australia) and then worked for three years as an environmental consultant on contaminated site assessment and remediation, before starting my PhD in identifying unknown toxicants in effect directed analysis at the Helmholtz Centre for Environmental Research (UFZ) in Leipzig, Germany. This was followed by six years as a postdoctoral fellow/scientist at Eawag—the Swiss Federal Institute of Aquatic Science and Technology—before moving to Luxembourg to start my own group in Environmental Cheminformatics (ECI) at the Luxembourg Centre for Systems Biomedicine (LCSB) within the University of Luxembourg. As a dual career couple in research, each of these moves has been a careful balance of career and family factors; we are the first couple to both receive ATTRACT Fellowships (in different years) from the Luxembourg National Research Fund (FNR) to establish our research groups in Luxembourg.

## What is your current research focus, and what are your plans for the future?

*NSC:* My research focus lies on computational chemogenomics. I am interested in the development, validation, and application of in silico tools for the identification of novel drugs and drug targets. With the increasing interest of the scientific community in artificial intelligence, I would like to explore how far these tools can take us, but also study their drawbacks and limitations. My short-term plan is to consolidate a research group in this field and disseminate the use of computational approaches in drug discovery research, particularly in Mexico and Latin America, where the use of these methodologies has not been widely extended. Thinking in the long-term, I would like to form strong collaborations with experimental groups, and why not, maybe even incorporate wet lab experiments into my group someday. *ELS:* Currently, as a research group, we are still focusing on the identification of unknowns, which is still a huge bottleneck in non-target screening in high-resolution mass spectrometry measurements (used for environmental, metabolomics, medical and exposomics applications, amongst many others). We are transitioning from method developments further into applications (exposome, biomedical, environmental, mixtures) [6, 7]. We are also still strongly focusing on improving data exchange (both Open and FAIR, *i.e.* findable, accessible, interoperable and reusable [8, 9]) in these fields, to alleviate several bottlenecks [10–12]. We also invest efforts into tackling some really tough cheminformatics challenges that are holding back environmental science and exposomics from making progress, such as UVCBs (materials of unknown or variable composition, complex reaction products or biological materials) [13]. In the future, I hope that our current efforts will soon solve current problems so we can move onto the next set of interesting challenges! However, there is still much to do with our current focus.

## Which obstacles did you encounter during your career, and what experiences have helped you get to where you are today?

*NSC:* I think that my career could be considered as a “common academic path” in some sense, since I spent most of it in academia. However, for me it was not common at all. I grew up in a lower-middle-class family, and as far as I know, I am the first in my family in pursuing a scientific career, so this has been a very uncommon path for my social context; I was expected to get a “real job” right after my bachelor’s degree, so in some sense I had to swim against the tide. Outside of personal matters, on the academic field, it is worth mentioning that my bachelor's degree had a total focus on the experimental part of medicinal chemistry, so while this represented a challenge when moving to the computational field, this experience also provided me with a critical thinking to know when and how to use computational tools. Some experiences for which I am truly grateful were the possibility of becoming an international student for some time, the chance of attending international conferences, and the opportunity to be involved in industry at Chemotargets SL, these experiences helped me a lot. Not only because I was forced to get by in a different language, or for the knowledge I acquired, but for the opportunity of get to know people from different parts of the world with distinct cultural and academic backgrounds as well as motivations and ways to see academic life. I must say that all this was possible thanks to the fact that I did all my studies at UNAM, because without its public education and the help of different scholarships, I would not have been able to get to where I am today.

*ELS:* Looking at my rather unconventional career path, several experiences have helped. Studying engineering and the three years as a consultant gave critical practical experiences that can often be missing in classic academic paths. Coming up through the environmental academic community, diversity was “normal”, audiences and conferences have been completely mixed, and I was rarely aware of being either female or a foreigner. This is often in stark contrast to the atmosphere in some other fields. However, it is possible to have a constructive and inclusive atmosphere even with extremely unbalanced numbers; I’ve experienced this in the past as the only female on mining sites, where I was totally at home and one of the team, and it would be great if this would apply everywhere. I am definitely grateful to the FNR, who gave both of us (my husband and I) the opportunity and resources to pursue our scientific careers, as it has been a huge challenge to unite family and career. This career support has been vital to help us get to where we are today as a research group (and family).

## What advice would you give to your younger self?

*NSC:* I have a couple: learn programming and do something with what you already know. With regards to the first point, programming is one of those things I wish I had known earlier, because it changed my life, and considering the computational power we have nowadays even in portable devices, we could achieve great things with that in almost every field. Regarding the second point, I consider myself to be a good student (easy learning, good grades, etc.); however, it took me a while to realize that it does not mean much if you do not do anything with that knowledge. I am not talking only about doing research; science dissemination and teaching others are good examples of what one can do from very early stages to pass on knowledge.

*ELS:* Gather data—no kidding, this has been very valuable advice I have been given, applicable to both life and research. Even if you have a strong gut feeling (pro or con), be patient and gather the data to make an informed decision before rushing in. It is time well invested.

## What is a current challenge you are facing that should not be a challenge in the near future?

*NSC:* As a user and developer of computational tools for drug discovery I can say that with the increasing number of new methodologies in this area, it becomes difficult to distinguish when and where to apply each of them, especially those involving the use of artificial intelligence techniques, which are often validated on different data sets and conditions. The establishment of “gold standards” for the thorough validation and comparison of these methodologies in different tasks is crucial, and something we need to address in the short term. Leaving science aside, one of the challenges I face as a Latin American researcher is the fact that funding is limited compared to high-income countries, which has a direct impact on the performance of our research groups. Our governments must understand that investment in research and development is a key component to accelerate economic growth in Latin American countries, otherwise we will remain stuck in a chicken-and-egg situation, as explained recently by Dr. Ana M. Valenzuela-Toro and Dr. Mariana Viglino [14]. *ELS:* We invest a disproportionate amount of our time extracting and curating extremely valuable chemical information (typically only either Name or Chemical Abstract Services (CAS) registry numbers or possibly both) from PDF files. We really need FAIR chemical data [10, 11] with more exact chemical information—these logistical issues are locking up so much knowledge and holding up so much research progress. From a more personal perspective, it is still disappointing that the career state/title of a female Professor or Principal Investigator (PI) is often underestimated or underrepresented in either casual scientific conversation (e.g., “where are you doing your PhD/postdoc”) or even in official communications and events (e.g., males are “Dr.” or “Prof.” and female “Ms.”). This also happens regularly to my fellow female PIs. It is extremely uncomfortable for us to have to be overassertive of titles, but caution is still needed to treat everyone consistently (all with titles, or no titles; no “role” assumptions), to help break down the implicit biases that have developed.

## What do you think the cheminformatics community could do to increase diversity and inclusion?

*NSC:* From a purely scientific perspective, keeping promoting open science is crucial, because it allows equal access to knowledge. Of course, this involves an economic cost (e.g., publishing in open access journals, attending to scientific meetings, etc.) that young students and researchers, especially those from developing countries may not always be able to cover. Looking for funding to support them through discounts or waivers for publishing, as well as bursaries for attending scientific meetings is something that the cheminformatics community must continue to do. On other side, considering the cultural and social underrepresentation of some groups in our actual society, efforts to promote their inclusion are needed. Journals publishing special issues focused on contributions from researchers related to these groups would contribute to increase their visibility for the whole scientific community. Good examples of this are the initiatives taken by the *Artificial Intelligence in the Life Sciences* journal, where special issues putting together contributions from female researchers or Latin American researchers have been launched. There is no doubt that there is still a long way to go on these topics, but it is good to see that we are making progress. 

*ELS:* I think that this article series is a wonderful initiative to start a discussion and exchange of ideas and experiences about diversity and inclusion, so that the next steps forward become clearer. I’m also a big fan of leading by example, since actions speak louder than words. I’ve always felt more comfortable myself in a diverse, constructive and inclusive environment and learn a lot from my very diverse team. I hope we as a research group can provide a comfortable home for researchers to learn about and work on FAIR, Open and diverse cheminformatics, and that we, as a community, can welcome a wide variety of experiences and opinions with open arms. I look forward to working with many individuals and groups in the cheminformatics community in the years to come.

## Imagine you won $10 M in the lottery—what would you do?

*NSC:* I would use most of the money to start a non-profit organization with the aim of supporting young people in difficult economic situations to pursue their studies and careers. I would like everyone who wants to, to have the same opportunities that I had; or to paraphrase Neil deGrasse Tyson, “I want that all the people with great ideas that could improve our world to have the chance to at least try”. I think education is one way to achieve this, so this would be my way to support it beyond teaching and research. 

*ELS:* I don’t really participate in lotteries so this is unlikely to happen to me (although these days, applying for funding seems to be a lottery…), but honestly, the way the world is right now, I would be tempted to donate the vast majority, if not all, to those who need it more than me. Otherwise, I would see this as a $10 M win for Open Chemical Science and this could be used to continue to fill knowledge gaps and develop open methods for the benefit of the environmental and cheminformatics communities.

## Photos

**Table:** 

	
﻿Norberto Sánchez-Cruz	Emma﻿ L. Schymanski

## Data Availability

Not applicable.
